# Sexual conflict and the Trivers-Willard hypothesis: Females prefer daughters and males prefer sons

**DOI:** 10.1038/s41598-018-33650-1

**Published:** 2018-10-18

**Authors:** Robert Lynch, Helen Wasielewski, Lee Cronk

**Affiliations:** 1 Department of Biology, University of Turku, Vesilinnantie 5, Turku, FIN-20014 Finland; 2 Department of Psychology, Arizona State University, 950S. McAllister Ave, Tempe, AZ 85287 USA; 3 Department of Anthropology, Rutgers University, 131 George Street, New Brunswick, NJ 08901 USA

## Abstract

Because parental care is expected to depend on the fitness returns generated by each unit of investment, it should be sensitive to both offspring condition and parental ability to invest. The Trivers-Willard Hypothesis (TWH) predicts that parents who are in good condition will bias investment towards sons, while parents who are in poor condition will bias investment towards daughters because high-quality sons are expected to out-reproduce high quality daughters, while low-quality daughters are expected to out-reproduce low quality sons. We report results from an online experiment testing the Trivers-Willard effect by measuring implicit and explicit psychological preferences and behaviorally implied preferences for sons or daughters both as a function of their social and economic status and in the aftermath of a priming task designed to make participants feel wealthy or poor. We find only limited support for predictions derived from the TWH and instead find that women have strong preferences for girls and men have preferences for boys.

## Introduction

Our current understanding of how resources are allocated to maximize the reproductive success of sons and daughters is based on Fisher’s principle of equal investment in the sexes^1^. Carl Duesing was the first to demonstrate that many sexually reproducing species produce an equal number of males and females because the total reproductive value of males and females is necessarily equal^2^. However, because sex allocation depends on the fitness returns to parental effort, selection will favor equal investment in sons and daughters only when the cost of producing each sex is identical. This argument is one of the best understood and most celebrated theories in evolutionary biology^3^ because it provides a framework for understanding the observed variance in sex ratios across species. In humans, although parental expenditure in each sex is expected to be equal, excess male mortality throughout the period of parental investment decreases the average costs of producing sons^1^. This is why most human populations are male- biased at birth^4,5^ but nearly equal among individuals who are sexually mature.

Although population-wide sex ratios are expected to be highly constrained due to frequency-dependent selection, evolution can favor deviations from Fisherian sex ratios if producing one sex has a greater payoff than the other in terms of the production of grand-offspring. Trivers and Willard, for example, hypothesized that natural selection should favor a parent’s ability to adjust offspring sex ratios according to their ability to invest^6^. The Trivers-Willard Hypothesis (TWH) relies on 3 critical assumptions: (1) that parental condition is correlated with offspring condition, (2) that offspring condition is correlated with condition in adulthood and (3) that condition differentially affects the mating success of each sex (e.g., males in good condition out-reproducing females in good condition and females in poor condition out-reproducing males in poor condition). Because these conditions are seen to hold for many species of mammals, Trivers and Willard argued that mothers in good condition should invest more in producing males while mothers who are in poor condition should invest more in producing females. In the years since it was proposed, the TWH has been subject to hundreds of tests, with mixed results and ongoing debates about such issues as the appropriateness of the species or population being studied, the timing of when such biases are expected to occur, and the validity of various measures of parental investment, parental condition, and offspring condition^7^.

In its original formulation, the TWH focused exclusively on maternal condition and sex ratios at birth in mammals. However, because most investment in human offspring occurs postnatally and comes from both parents, many of the most convincing studies of TW effects in human populations have also analyzed care by fathers and have included measures of sex-biased investment after birth^8^. For example, Cronk collected data on sex biases in parental investment among the Mukogodo of Kenya, who are at the bottom of a regional hierarchy whereby men often either do not marry or have delayed marriage, and, as a result, have lower mean reproductive success than the women. He found that, on average, Mukogodo parents wean daughters later, spend more time nursing and holding daughters, remain physically closer to daughters, and are more likely to take their daughters than their sons for medical care, all of which results in better growth performance for Mukogodo girls, than boys, and a female bias in the sex ratio of children aged 0 through 4 years^9–11^. Meanwhile, a study of parents in the United States, using self-reports and diary data, found no evidence that parents’ socioeconomic status is associated with biased investment in either sex^12^. These contradictory results point to another potential pitfall that many studies purporting to test the TWH encounter by failing to sufficiently distinguish between physiological, psychological and behavioral outcomes. There is an abundance of evidence that there are often large discrepancies between stated offspring sex preferences and parents’ actual behavior toward offspring^3–15^ and it is often the case that studies using behavioral measures are more likely to provide support^16–18^. Therefore it is crucial that researchers distinguish between physiological outcomes (e.g., factors that hinder the implantation of embryos), preferences implied by behavior (e.g., time spent nursing) and those assessed psychologically (e.g., self-reports or implicit preferences) that depend on whether the offspring is male or female. Other studies have further complicated attempts to reach a scientific consensus by showing that offspring condition, regardless of sex, and overall family income, can affect investment decisions. Increasing a family’s resources, for example, has been shown to result in a shift from concerns with efficiency to concerns with equity^19^ and some studies have shown that poor households reduce investment in children who are at high risk but then increase investment in those children once the families obtain more resources^20,21^.

Much of the confusion and inconsistent results over TWH research has also resulted from a failure to distinguish between when it is optimal for parents, especially mothers, to bias offspring sex ratios vs. when it is optimal for them to bias investment by sex. Although many researchers have considered sex ratios biases and post conception investment to be on the same continuum, seeing them as similar ways of optimizing the allocation of parental investment, this is not always the case. The distinction relies on the third assumption of the TWH — that condition differentially impacts the reproductive success of each sex6. Veller *et al*.^22^, for instance, have shown that it makes sense to produce male biased sex ratios when the mother is in good condition because the absolute returns on producing sons with high fitness is higher than it is for producing daughters with high fitness. This is because the fitness value of males increases more in response to improved maternal condition. However, this is not necessarily true of post-conception investment biases because they depend on marginal returns on investment. Here investment should be biased towards whichever offspring improves parental fitness more per unit invested and this is not necessarily linked to the overall fitness value of the offspring^12^. If mothers who are in poor condition receive higher fitness returns per unit invested than mothers who are in good condition they may be expected to bias investment towards sons. In other words, whenever male fitness increases faster with condition than does female fitness (i.e. the male fitness function has a steeper slope), the fitness returns on sons will always be greater and parents will have greater marginal gains (per unit of investment) by investing in males regardless of their own condition. Several studies provide support for making a distinction between when parents are expected to bias sex ratios vs. when they are expected to bias investment. A meta-analysis of mammalian sex ratios, for example, showed that studies analyzing sex ratios around conception showed nearly unanimous support for the hypothesis that mothers in good condition bias litters towards sons^23^. Meanwhile a broad review of the literature surveying Trivers-Willard effects on postnatal parental investment in humans yields somewhat less consistent results, with studies that operationalized key variables in more appropriate ways and those which were conducted on populations that better conformed to the assumptions of the hypothesis tending to show more support for it 7.

To date, most evolutionary hypotheses on sex-biased parental investment have assumed that resource constraints affect both parents in exactly the same way, and that under certain conditions, mothers and fathers will converge on the same investment biases and preferences. This is despite considerable evidence showing that fathers prefer sons and mothers prefer daughters^24^. Which parent controls and distributes resources has also been shown to influence outcomes for boys and girls: in a small-scale horticulturalist society where food is not always abundant, maternal control of resources was positively associated with increased BMI of daughters (necessary for gestation and lactation) relative to sons^25^. In the United States, fathers are also more likely to be present in the home if their child is male^26^, and male offspring reduce the risk that fathers’ will initiate divorce by approximately 9%^27^. Another study found that American men work more and harder following the birth of sons but not of daughters^28^. Meanwhile, American mothers who head the household after a divorce pay more attention to their daughters than to their sons^26^. Some researchers have argued that these biases are adaptive because children are more likely to benefit from investment from their same-sex parent who can better help them by providing information about their future sex roles^24,25^. Therefore, these sex biases may be expected to work in both directions, such that parents are not only primed to transmit sex-specific information to their same-sex offspring, but that offspring are also predisposed to learn from their same-sex parents^29^. The sex of the parent has even been shown to affect the heights of same- and opposite-sex offspring, which may indicate biases in PI. A study conducted in Brazil, the United States and Ghana showed that mothers’ level of education was positively correlated with the height and health of their daughters, but not their sons, while father’s educational attainment was positively correlated with the height of their sons^29^.

Evolution can select for parents who favor same-sex offspring when the evolutionary interests of males and females diverge. On a genetic level, whenever males and females have different optimal outcomes for traits that are expressed in both sexes, intralocus sexual conflict is expected^30^. Intralocus sexual conflict occurs when genes that benefit one sex are detrimental to the other^31^, which can affect the transmission of genetic fitness to same- and opposite-sex offspring. In other words, fathers with ‘good genes for males’ may produce sons with high fitness but will produce daughters with low fitness. At the same time, mothers with ‘good genes for females’ may produce daughters with high fitness but sons with low fitness. This disruption of the transmission of genetic quality to same and opposite sex offspring^30^ violates one of the crucial assumptions of the TWH — that parental condition is positively correlated with offspring condition. The uneven transfer of fitness to same- and opposite-sex offspring might also be expected to affect selection on parental investment and even sex ratios. In a species of flour beetles, for example, low-fitness females produced more sons while high-fitness females produced more daughters^32^. Sexual conflict can therefore alter optimal investment strategies such that sex-biased PI may depend not only on the condition of the parent but also on their sex. The evolutionary importance of intralocus sexual conflict was not understood when Trivers and Willard wrote their paper in 1973, and understanding the interactions between the condition of mothers and fathers and the condition of sons and daughters may help to shed light on some of the inconsistent and contradictory findings of TWH research over the years.

Frequency dependent constraints on local and population-wide sex ratios (Fisher’s principle), the disparate benefits of transmitting culturally learned traits to same- and opposite-sex offspring, the uneven benefits of sexually antagonistic genes transferred from mothers and fathers to sons and daughters, the influence of cultural and societal norms regarding which sex is favored and the varied impact of resources on the fitness of sons and daughters all conspire to complicate ‘optimal’ PI decisions for mothers and fathers. Detecting these effects, or even knowing what to expect, can be difficult when the expected outcome of one strategy (e.g. wealthy mothers should favor sons) conflicts with or masks another (e.g. mothers with good genes should favor daughters).

## The Current Study

One of the most important questions that remains in the literature on the Trivers-Willard Hypothesis is the nature of the proximate mechanism that allows parents to bias their investment. Although there are several good experimental studies showing physiological triggers, including increasing the fat content in diet^33^, inducing diabetes^34^ and decreasing the circulating levels of glucose^35^ in mice, we are aware of only two studies that include experimental manipulations of potential proximate psychological mechanisms. Mathews^36^ attempted to prime childless participants to feel “in poor condition” by having them think about their own mortality, and found no impact of this prime on the desired sex ratio of participants’ future children. However, it is unclear why thoughts about one’s own mortality would lead to a sense that one is in poor condition as a parent. A more promising approach was taken by Durante *et al*.^37^ who used slides depicting either an economic upswing or an economic recession to prime participants on Amazon Mechanical Turk (MTurk). Participants who saw the slide depicting the effects of a recession reported preferences favoring investments in daughters including a stronger desire to give a hypothetical US Treasury bond to a daughter than to a son and a willingness to bequeath more assets to a daughter than a son in their will.

Like Durante *et al*.^37^, we attempted to trigger a Trivers-Willard effect by priming MTurk participants with IP addresses in the United States to feel either poor or rich. We also collected survey and behavioral data on participants’ preferences and backgrounds, offered them the choice of donating to a charity that benefits either both or girls who participants were asked to think of as their own, and had them take an Implicit Attitude Test (IAT) regarding their feelings about boys vs. girls. Previous research has suggested that parental investment decisions may be influenced by conditions faced by the parent^6^ as well as those experienced in childhood^38^. We predicted that individuals who were primed to be poor would P1a) prefer to adopt daughters, P1b) donate more money to a charity that helps baby girls than to one that helps baby boys, P1c) show implicit preferences for girls and P1d) express explicit preferences for daughters. We also analyzed the relationship between both childhood and adult socio-economic condition on offspring sex preferences. We predicted that individuals who had low social status either as children or as adults would P2a) prefer to adopt daughters, P2b) donate more money to a charity that helps baby girls than to one that helps baby boys, P2c) show implicit preferences for girls and P2d) express explicit preferences for daughters.

## Results

### Descriptive statistics

Overall, 347 females (coded as 0) and 423 males (coded as 1) with a mean age of 36.3 years old, 316 (40%) of whom were either currently married or had been divorced and 502 (65%) of whom had already had at least one child completed the survey and were paid on Amazon Turk. The survey was generated through Qualtrics software, which requires subjects to complete each section before they are able to continue, and all 770 subjects who completed the survey were used in these analyses. Participants reported their incomes as follows: (1) less than $20,000 (N = 140), (2) $20,000–$45,000 (N = 263), (3) $45,001–$70,000 (N = 185), (4) $70,001–$100,000 (N = 116) and (5) greater than $100,000 (N = 66). The median response to the question about income was the same as the mode, i.e., $20,000–$45,000. Most participants reported that they either were currently enrolled in school (N = 76), had graduated from high school (N = 86), had attended some college (N = 216), or had graduated from either a two-year (N = 70) or a four-year college (N = 311). Smaller numbers reported that they had less than a ninth grade education (N = 1), some high school (N = 2), a master’s degree (N = 62), a professional degree (N = 15), or a doctoral degree (N = 7). Most participants reported that their parents had graduated from high school (N = 189), a two-year college (N = 75), or a four-year college (N = 210). Smaller numbers reported that their parents had less than a ninth grade education (N = 12), some high school (N = 24), a master’s degree (N = 88), a professional degree (N = 14), or a doctoral degree (N = 19). The mean response to the question about perceived relative status was 4.96, which is slightly below the center of the ladder (5.5). Most rated their health as good (N = 239), very good (N = 319) or excellent (N = 120), with relatively few rating it as either poor (N = 11) or fair (N = 81). Most participants were single (N = 427), some were married (N = 276), a few were divorced (N = 53), and very few were either separated (N = 6) or widowed (N = 8). In answer to the question about adoption, more participants elected to adopt ‘the girl’ (N = 442) rather than ‘the boy’ (N = 328). Implicit Association Tests were completed by 307 female and 375 male participants. Because the IAT test required subjects to access a separate website and Qualtrics software was unable to verify whether or not they had successfully completed it in order to permit them to proceed with the survey, 88 participants did not complete this task. There also may have been some confusion about how to access the website. The mean score for all participants on the Implicit Attitude Test was −0.137 indicating, on average, an overall preference for girls.

### The effect of priming individuals to feel rich or poor on offspring sex biases (Predictions 1a–1d)

Males who were primed to feel wealthy donated significantly more to charities supporting girls. Overall, however, the experimental prime had very little impact on any of these results and statistical significance did not survive post-hoc tests for multiple comparisons (e.g. Bonferroni correction) (see Table 1).

**Table 1:** Parameter estimates for predictors in top ranked ranked models (lowest WAIC score) for males, females and full sample.

Top Models										
DV	Predictor	Males			Females			Full model		
		B	SE	p-val	B	SE	p-val	B	SE	p-val
Adoption	*(Intercept)*	0.37	0.09	***	0.56	0.11	***	0.38	0.11	***
	*Sex*							0.17	0.04	***
	*Childhood poverty*	0.05	0.02	**				0.006	0.02	0.73
	*Adult poverty*				0.06	0.04	0.08^†^	0.05	0.03	0.09^†^
	*Perceived status*	0.02	0.01	0.07^†^				0.02	0.01	0.15
	*Education*				−0.04	0.02	0.05*	−0.02	0.01	0.11
	*Sex X Childhood poverty*							0.04	0.02	0.09^†^
Donations	*(Intercept)*	−4.8	1.7	**	0.50	0.03	***	0.92	1.6	0.56
	*Sex*							−6.3	2.25	***
	*Childhood poverty*	0.01	0.005	0.07^†^				−0.001	0.006	0.82
	*Adult poverty*							0.004	0.01	0.71
	*Perceived status*	0.02	0.005	***				−0.001	0.006	0.83
	*Education*				−0.01	0.006	0.08^†^	−0.003	0.005	0.58
	*Parents education*				−0.009	0.005	0.06^†^	−0.008	0.003	0.02*
	*Rich prime*	−0.02	0.01	0.03*				−0.02	0.007	0.01*
	*YOB*	−0.002	0.0001	**				0.00	0.00	0.76
	*Sex X Childhood poverty*							0.014	0.008	0.07^†^
	*Sex X Perceived status*							0.02	0.007	**
	*Sex X YOB*							0.003	0.001	***
IAT	*(Intercept)*	0.11	0.02	***	−0.44	0.02	***	−0.36	0.05	***
	*Sex*							0.55	0.03	***
	*Adult poverty*									
	*IAT order*				0.11	0.05	0.03*			
	*Parents education*							−0.017	0.01	0.09^†^
	*Rich prime*							−0.04	0.02	0.09^†^
Preferred SR	*(Intercept)*	0.54	0.05	***	0.48	0.01	***	0.48	0.01	***
	*Sex*							0.04	0.02	0.02*
	*Education*	−0.02	0.009	0.04*						
	*Parents education*	0.01	0.007	0.04*						
	*Rich prime*							−0.02	0.01	0.14
	*YOB*	0.002	0.001	0.15						

For predictor variables, higher values equal higher status (parents education, education, perceived status, childhood poverty, adult poverty, income, rich prime) and for sex (0 = female, 1 = male). For outcome variables higher = preference for boys. Significance levels ^†^p < 0.1, *p < 0.05, **p < 0.01, ***p < 0.001.

### The effect of the participant’s condition on offspring sex biases (Predictions 2a–2d)

**Figure 1:**
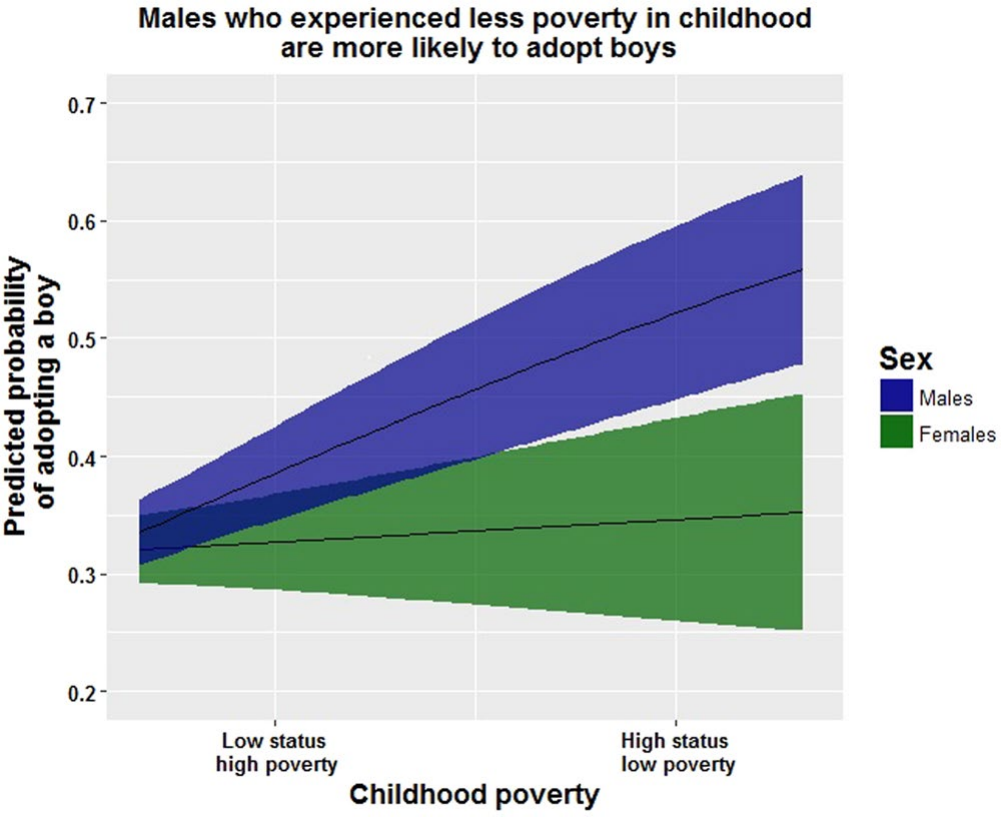
The preference for adopting a boy is lower for females than for males across all conditions. Males who experience more poverty in childhood are more likely to express a preference to adopt females. The plot holds all the other variables (see Table 1) in the top models constant (i.e. holds them at their mean values) across each level of poverty in childhood (Shading around line is 95% CI).

**Figure 2:**
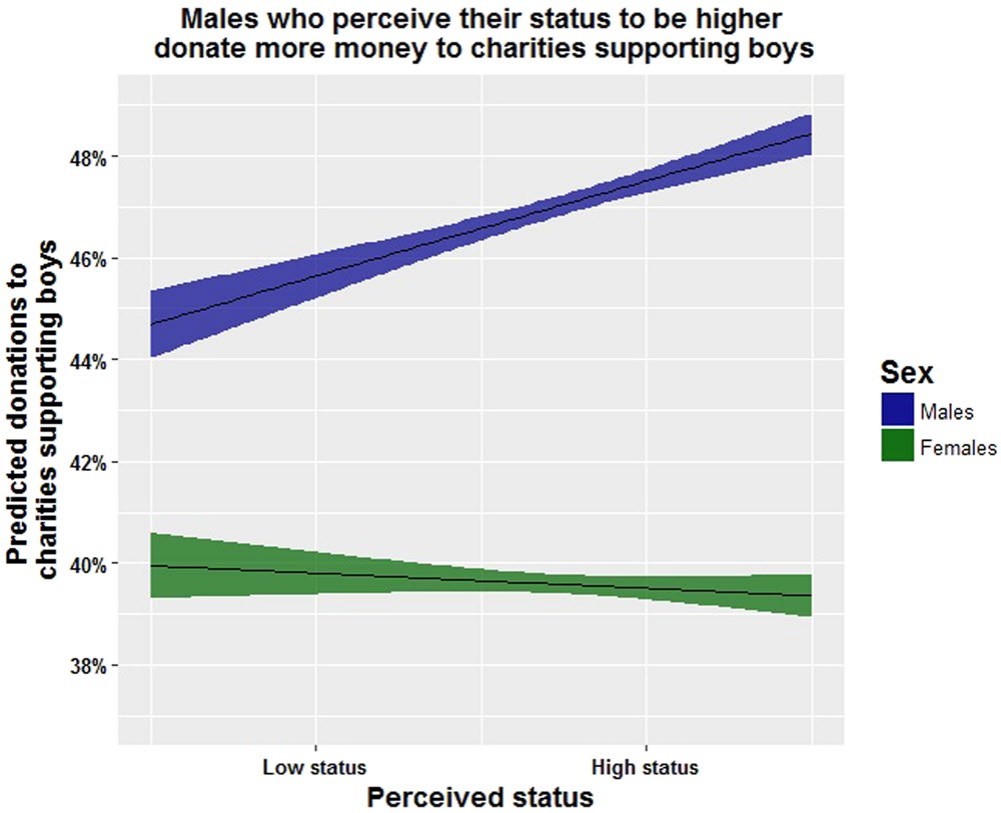
Both sexes, but especially females, donated more to charities supporting girls. Males who reported higher perceptions of their own status donated more to charities supporting boys.

Among male participants, lower childhood poverty predicted a preference to adopt males (Fig. 1), higher perceived status predicted more donations to charities supporting boys (Fig. 2), and younger males donated significantly more to charities supporting girls (Table 1). The participants’ own education and the participants’ parents’ education had opposite effects on preferred sex ratios among males: higher education of the participant predicts a preference for a female-biased sex ratio and higher education of the participant’s parents predicts a preference for a male-biased sex ratio. Again, however, none of these results survive post-hoc tests for multiple comparisons.

### Model selection

We used candidate sets of all the combinations of the predictor variables to model each of the four dependent variables described above (see Methods) in a Generalized Linear Model regression in R Studio 3.4.1. We fitted models with the package ‘lme4’ and used the ‘MuMIn’ package to fit all combinations of the predictor variables.Because the strongest correlations between any of the candidate predictor variables were between ‘Perceived relative status’ and ‘Income’ (r = 0.48) and between ‘Parent’s education’ and ‘Education’ (r = 0.29), we do not think that multicollinearity would adversely affect our models. Therefore we allowed all predictors to be entered into each model and ranked them by Akaike’s Information Criterion Score ultimately using all variables within 2 AICc units of the top-ranked model and averaging across them by their weight (see Supplementary Materials Tables S1:S12). The final predictor variables used were considered to be ‘informative’ and were seen as being the most useful in striking a balance between model complexity and overfitting^39^. See Supplementary materials Tables S1–S12 for all the top models, their AIC scores and rankings for males, females and the whole sample for each of the 4 dependent variables used. We evaluated model performance by calculating the area under the curve (AUC) of the receiver operating characteristic (ROC) for each of the top models^40^. The AUC evaluates a model’s performance by indicating how well the model predicts a participant’s response to the dependent variable. An AUC value of 1.0 indicates perfect predictability, and a value of 0.5 indicates the model’s predictability is equal to random. We considered values with 95% Confidence Intervals (CI’s) that did not overlap with 0.5 to be reasonable models^41^.

### Models

#### Adoption

Overall, females strongly preferred to adopt girls (65.7% preferred to adopt a girl, S.E. = 2.5%) and males showed no preference for either boys or girls (51.6% preferred to adopt a girl. S.E. = 2.4%) (see Table 2). A preference for adopting boys among men was significantly predicted by lower childhood poverty (Table 1 and Fig. 1). Among women, lower adult poverty predicted adoption preferences for boys and education level predicted a preference for adopting girls. Neither result, however, was statistically significant at the p < 0.05 level.

**Table 2:** Percentage of males and females who prefer to adopt boys, percent donated to a charity benefiting boys vs. girls, implicit association test score (positive = boy preference) and preferences for a male biased sex ratio.

	Males	Females
**Boy preferences**		
Adoption	48.4%	34.3%
Donations	46.2%	38.9%
IAT score	0.11	−0.44
Preferred SR	53%	49%

#### Donations

Females and males both gave more money to charities supporting baby girls than to charities supporting baby boys, but females gave substantially more (mean = 61.1 cents, S.E. = 0.7) to girls than did males, who gave 53.8 cents (S.E. = 0.8) to girls (Table 2). The prime seemed to affect only males, such that males who were primed to feel rich were significantly more likely to donate to the girl’s charity. For males who were primed to feel poor, the mean donation to charities supporting girls was 52.2 cents (S.E. = 1.5); males in the control group donated 50.8 cents (S.E. = 1.4), and males primed to be rich donated 58.5 cents (S.E. = 1.4). No differences were seen between treatments on the donations to females (Poor: 59 cents, Control 60.5 cents, Rich: 61.1 cents). Males who had higher perceived status gave significantly more to charities favoring boys (Fig. 2). Lower childhood poverty was a suggestive but non-significant predictor of donations to boys among males. Among females, education and parents’ education were non-significant predictors of donations to charities supporting girls (Table 1).

#### Implicit Association Test

**Figure 3:**
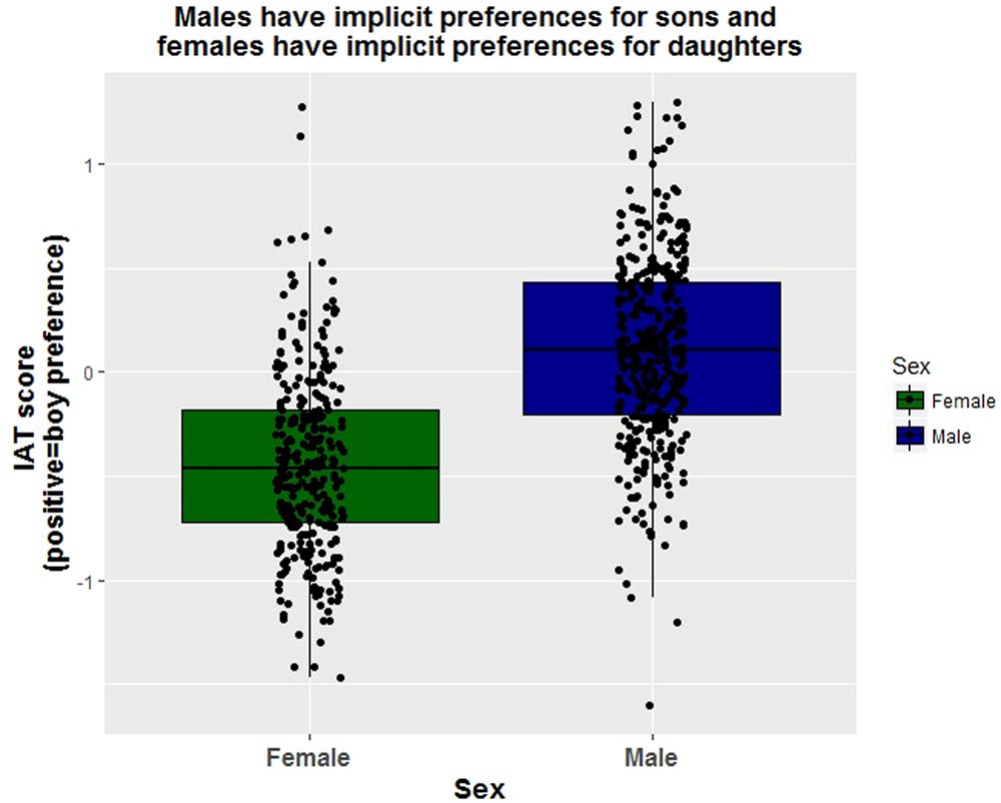
Both sexes showed implicit preferences for same sex children but females showed a stronger preference than males.

Overall, participants showed an implicit preference for girls (mean = −0.137, S.E. = 0.018), but females had a stronger implicit preference for girls (mean = −0.438, S.E. = 0.002) while males showed a slight preference for boys (mean = 0.11, S.E. = 0.022) (Fig. 3 and Table 1). The order of the IAT test also affected the results among females such that women who took the IAT test before they took the survey were more likely to show an implicit preference for boys (Table 1). None of the other independent variables appeared to have an impact on these results.

#### Preferred sex ratios

**Figure 4:**
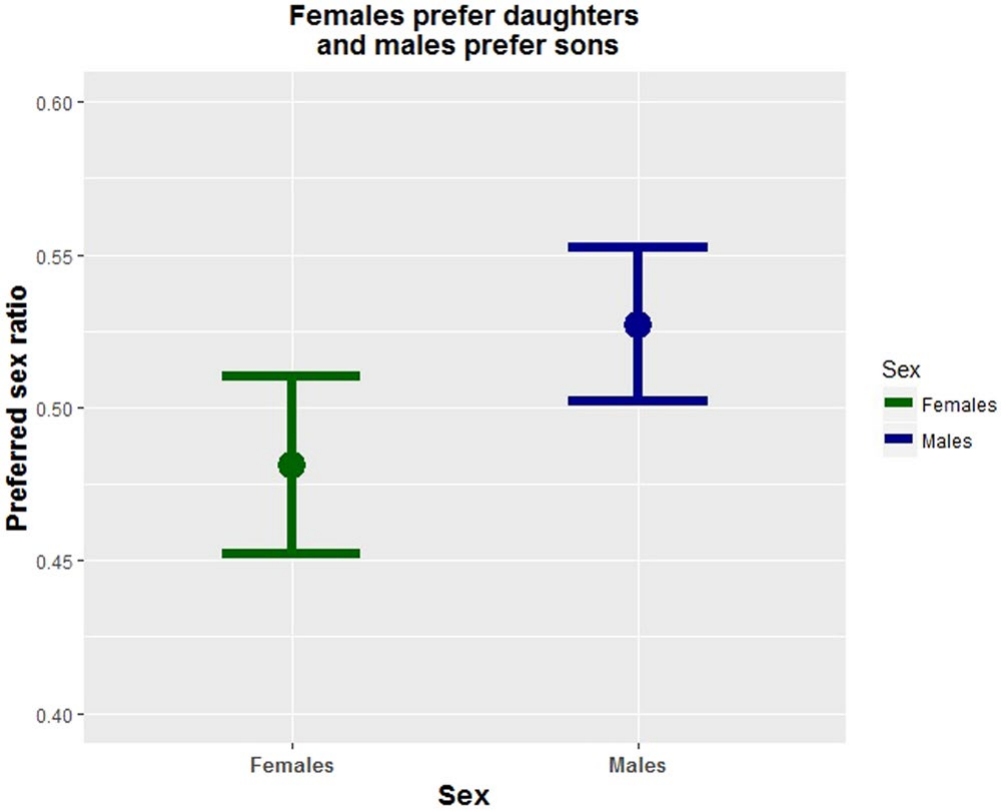
Each sex showed a weak, but statistically significant explicit preference for same sex offspring.

Overall, individuals expressed no preference for having daughters or sons (mean preferred sex ratio = 0.51, S.E. = 0.008) but within sexes, females expressed a slight preference for daughters (mean preferred sex ratio = 0.48, S.E. = 0.01) and males expressed a slight preference for sons (mean preferred sex ratio = 0.53, S.E. = 0.01) (Fig. 4). For males, education of the participants and their parents affected preferences oppositely such that the education of a male’s parents was a barely significant predictor of a son preference while their own education was a barely significant predictor of a daughter preference. No notable effects were observed for female explicit offspring sex ratio preferences (Table 2). The dependent variables were all positively correlated (Table 3).

**Table 3:** Covariance amongst the dependent variables.

	Adoption	Donations	IAT score	Preferred SR
**Relationships amongst the DV’s**				
Adoption	—	0.25	0.36	0.17
Donations	0.25	—	0.21	0.28
IAT score	0.17	0.21	—	0.17
Preferred SR	0.36	0.28	0.17	—

## Discussion

There was no convincing evidence for any of our a priori predictions. The only results that provide any support for the TWH at all are that males who grew up in poverty and males with lower perceived SES prior to any priming condition were more likely to choose to adopt girls. However, neither of these results survive statistical tests for multiple comparisons (e.g. a Bonferroni correction compensating for the fact that more than one hypothesis was tested by redefining a ‘significant’ p-value as one that is less than 0.05/the number of hypotheses tested). Although these results provide only suggestive support for TWH, it is worth noting that adoption preference falls under the more general conditions under which TW effects are expected (i.e. conditions that depend on the fitness value of offspring), rather than the more limited conditions expected to trigger sex biased investment (i.e. conditions that depend on the marginal fitness returns per unit of parental investment)^22^. However, our experiment did uncover an unpredicted and interesting association between participants’ own sex and their preferences for girls and boys, with females exhibiting a strong preference for girls and males exhibiting a weaker preference for boys. Female participants showed a strong preference for adopting girls, donated far more to charities supporting girls rather than boys, scored much lower on the Implicit Association Test (i.e. implicit preference for girls), and preferred female-biased offspring sex ratios. Males, meanwhile, showed no significant preference for adopting daughters vs. sons, a modest preference for donating to charities supporting girls, a slight implicit preference for boys and a slight explicit preference for a male-biased offspring sex ratio (see Table 1). We discuss these results within the theoretical context of sex-biased PI.

### Constraints on resources

Why should females across all experimental conditions and low-status males prefer daughters? Focusing on constraints on resources rather than on the sex or the condition of the parent offers one way to understand these results and put them in a larger theoretical context. Economists studying sex biased investment in offspring often focus on maximizing the socioeconomic benefits to the household^19,42^ and some have argued that constraints on resources produce an unequal optimal allocation of goods and services within the household^43,44^. Evolutionary theorists, meanwhile, have focused on fitness benefits^6,45–48^ and have primarily tried to explain how parental condition can affect optimal investment in sons and daughters. The TWH, however, was originally about parental ability to invest in offspring rather than parental condition6 and parental condition was seen as a proxy of parental ability to invest. If males and females who share a common household have differential access to ‘shared’ resources, and if increasing this access induces a parent to bias investment towards sons, while decreasing this access induces a parent to bias investments towards daughters, we may then be able to better understand why mothers and fathers in the same household might differ in their investment in daughters and sons. In other words, by focusing on sex differences in access to household resources, we may gain insight into why mothers and fathers in the same household might differ in their investment in daughters and sons. Godoy *et al*.^25^, for example, argued that in some cultural and social contexts there are systematic sex differences in access to household resources between men and women and hypothesized that the sex facing more resource constraints will exhibit a stronger preference for girls while the sex facing fewer constraints will show a preference for boys. In other words, when resources are pooled and one sex has more access to them than the other, we may expect that offspring sex preferences will be driven by both the sex of the parent (owing to differential access to ‘shared’ resources) and their condition (total shared resources). If this interpretation is correct, then our finding that females exhibit a preference for daughters may be the consequence of females having lower access to shared resources than males. Similarly, our finding that lower-status males also exhibit a preference for daughters may be the consequence of lower-status males facing higher constraints on their ability to invest.

### Sexually antagonistic genes

**Figure 5:**
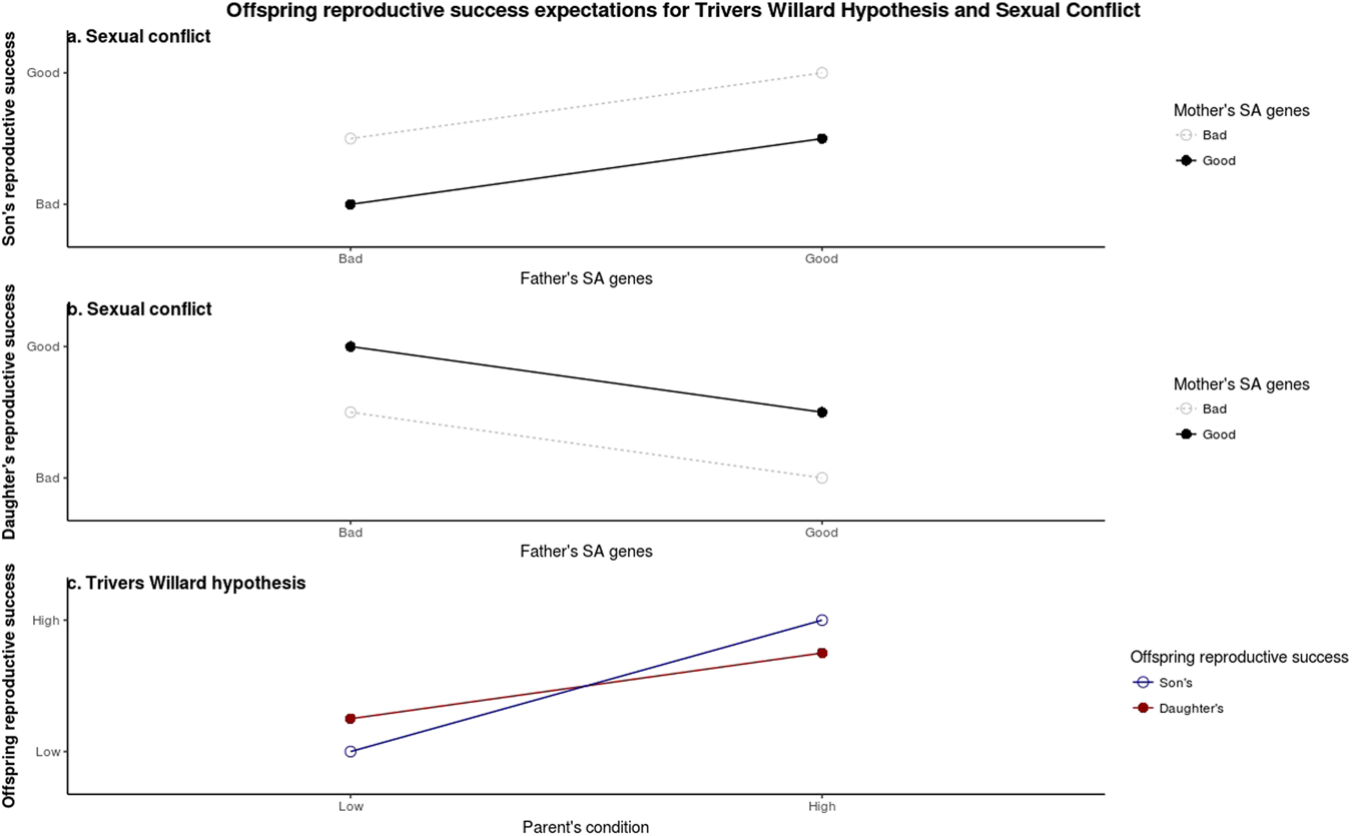
Theoretical predictions made by sexual conflict and TWH for the reproductive success of sons and daughters as a function of mother and fathers sexually antagonistic genes (**a**) and (**b**) [adapted from^71^] and the condition of both parents (**c**) [adapted from^9^].

Intralocus sexual conflict, which has now been confirmed in humans^49,50^, may also help to explain these results. If male condition is positively correlated with male genetic quality, and if some proportion of the genes that affect male condition are sexually antagonistic (i.e., have opposing fitness effects in males and females), then fathers with poor genes will produce low quality sons and high quality daughters. In this situation, the predictions made by sexual conflict theory and the TWH are the same — males with poor genes and males who are in poor condition will invest more in daughters. In contrast, when males either have good sexually antagonistic genes (i.e. good genes for sons) or when they are in good condition, the Trivers-Willard hypothesis and sexual conflict theory respectively predict that they will invest more in sons. For females, however, the predictions made by the two theories conflict. For females with poor genes, sexual conflict predicts that they would be better off investing in sons. If, however, these poor genes result in a mother being in poor condition, the TWH predicts they should invest in daughters. This situation is exactly reversed for females who are in good condition due to their having good genes. Here sexual conflict theory predicts greater investment in daughters for females (good SA genes) while TWH predicts greater investment in sons (good condition). Whenever genetic quality and condition are positively correlated this can produce opposing selection pressures on females (see Fig. 5 for the theoretical predictions made by sexual conflict and the TWH).

Although this study does not directly measure the genetic quality of participants, we do find limited confirmation for preferences emerging from sexual conflict. Overall, our finding of a preference for girls on all four dependent variables could be attributed to the low status of MTurk workers relative to the population as a whole. MTurk workers tend to be educated but have low incomes (median income between 20,000 *and* 30,000)^51^. Our participants reported incomes in that same range (20,000–45,000), which puts them in the bottom 35% of the United States population. If true, then these daughter preferences for both males and females are consistent with the TWH. However, if sexual conflict affects these preferences and if most of our participants have found themselves in poor condition (low SES), but many of them have good genes, then we might expect that the males who have good genes but who are in poor condition will face a conflict. These males will be pulled by TW effects to favor daughters, but by good genes to favor sons. This conflict may help to explain the more moderate preferences for daughters exhibited by males in our study. On the other hand, the poor socio-economic condition of the females in our sample will push them towards favoring daughters (TWH) while those with good genes will also favor daughters. In this case all that we need to assume to explain these results is that all (or most) of our sample was in poor condition but only half of them had poor genes. If this assumption is true, then we would predict that daughter preferences will be stronger amongst females. This potential for conflict between the TWH and sexual conflict theory may also help to explain some of our more peculiar results. For example, if more educated women who come from more educated families have better genes but have found themselves in relatively poor condition we might expect these results, i.e., that they should prefer to both adopt girls and donate more to charities supporting girls (see Table 1).

### Cultural explanations

Parental investment patterns have been changing rapidly in developed countries like the United States over the past few decades and some evidence indicates that, overall, parents now invest more in daughters than they do in sons^52^ and prospective couples are 45% more likely to express an interest in adopting daughters over sons^53^. Another study showed that since 2008 there has been a sharp decrease in the likelihood of native-born Americans having another child after the birth of a daughter^54^ suggesting either an increase in preferences for daughters or a decrease in preferences for sons. Therefore these results showing overall preferences for daughters may reflect the cultural impact of parental sensitivity to increasing economic prospects for females in Western, industrial societies.

Hazan and Zoabi have suggested that, if parents are attempting to maximize returns on human capital (e.g., household income), then, as the returns on human capital increase, the relative advantage of females in education also increases, which in turn triggers more investment in daughters^55^. Because in the United States girls outperform boys in school and are far more likely to attend college, the expected return on investment for daughters is rapidly increasing, which may account for the overall girl preference in our sample. In Iceland, which is widely considered one of the most gender neutral countries on Earth, girl preferences are strong^56^, which suggests that, as opportunities increase for girls and decrease for boys in the United States, offspring sex preferences may follow suit. There is also some evidence that, although overall parents tend to express preferences for their same sex offspring, fathers are increasingly likely to prefer daughters as genders roles have changed (e.g., girls are increasingly more likely to play sports)^57^.

### Caveats

Our failure to find stronger support for the TWH may be due to a disconnect between our study design and the likely nature of the Trivers-Willard psychological mechanism. Given that the selection pressures that would have favored the evolution of a Trivers-Willard mechanism have existed for far longer than our species and given that conscious, deliberative thought is, in evolutionary terms, a new aspect of our psychology, it is likely that any Trivers-Willard psychological mechanism is ancient, deeply rooted, and largely unconscious^7^. The idea that many parenting decisions may not involve conscious thought is supported by the frequency with which researchers have found mismatches between actual parental behavior and parents’ stated offspring sex preferences (reviewed in:13–15). Using the IAT test, which is often viewed as a way to circumvent introspection, decrease the mental resources available to produce a deliberate response, and reduce the role of conscious intention^58^ was our attempt to reduce the effects of conscious deliberation on our results. For the same reasons, we also deliberately avoided asking subjects whether they preferred sons or daughters after the prime and instead simply asked them to donate to a charity and to allocate their donation between boys and girls. Importantly, this is not simply a measure of stated preference but is a measure of actual behavior. It is also worth noting that latency times on implicit association tests have also been positively correlated with actual behavior^59,60^. Nevertheless, we acknowledge that it is far from clear how any of these processes enter conscious awareness, and we realize that alternative approaches may be better designed to avoid triggering conscious deliberation about which sex to favor.

Another important limitation of our study is the generalizability of these results. As we mentioned previously, MTurk workers are not a nationally representative probability sample of the United States. Therefore, in the strictest sense, these results are representative only of Amazon Turk workers. Nevertheless, analyses of the characteristics of MTurk workers show that they meet or exceed psychometric standards of published research (e.g. completion rates or test-retest reliabilities) and are significantly more diverse and more representative than those of college populations, internet-based samples^61^, or in-person convenience samples — the modal sample in published experimental political science journals - but less representative than subjects from national probability samples^62^. Another issue concerns the fact that participants were self-selected in the sense that they chose whether or not to participate in the study. However, we do not feel that this limits our ability to interpret our results. Although it is true that MTurk workers decide for themselves whether to participate, they do so without any foreknowledge of what the project is about. Furthermore, because this study employed an experimental design in which participants were randomly assigned to groups, random sampling is not necessary to obtain meaningful and interpretable results. This is because convenience sampling of participants such as MTurk workers does not threaten the internal validity of experiments in which there is random allocation of the sample members. Our random assignment of participants to one of three groups (control, rich prime and poor prime) suggests that any systematic differences in outcomes between treatment groups was due to differences in treatment and not to differences in some other unknown characteristic resulting from self selection or biased sampling.

### Implications

These results may also have implications for rising income inequality and intergenerational social mobility. A recent study using the tax records of 40 million Americans between 1996 and 2012 showed that the single best predictor of lower intergenerational social mobility was having a single or divorced parent^63^. Because most of these single parents are females^64^, and females prefer daughters, we might expect even lower reduced intergenerational mobility for the sons of these single mothers.

## Conclusion

Strong frequency-dependent selection on optimal investment and allocation in the sexes driven by Fisher’s principle of equal investment in the sexes1 means that deviations from these ratios are expected to be subtle and extremely difficult to detect. Therefore, we should not expect, and should actually be suspicious of, strong effects on sex-biased investment for both statistical^65^ and theoretical3 reasons. The Trivers-Willard hypothesis^6^, sexual conflict^31^, economic models of sex biased PI^25^, and cultural practices^66^ can all generate different predictions for optimal parental investment strategies. One effect (e.g., sexual conflict) can also often mask the effects of another (e.g., TWH). For example, a male who has low socioeconomic status and good genes is expected to produce sons with better genes than his daughters; but, owing to his low socioeconomic status, he may be better off investing in his daughters, even though they are predicted to have worse genes than his sons. Results of this study contribute to the complex, and often contradictory, literature on sex-biased investment and preferences in humans.

## Methods

Participants were recruited on Amazon Turk and were asked to complete a Qualtrics survey and an implicit association test [IAT]^67^ online. Although subjects chose whether or not to participate, they did not know what the task would involve and only saw that it was an ‘online survey’ and saw that they would be paid $2.00 for completing the task. The Qualtrics survey design template and Amazon Mechanical Turk oversight allowed for sufficient control over who actually completed the survey and tests so that every worker who completed the survey and was paid was used in these analyses. Thirty individuals started the survey but did not complete it and their responses were discarded. At the beginning of the survey, participants were randomly assigned to one of three groups: (1) viewed an experimental prime that was designed to make them feel wealthy, (2) viewed an experimental prime that was designed to make them feel poor (see Experimental prime below) or (3) was not primed and were assigned to a control group. The tasks were counterbalanced such that half the participants took the IAT test before the prime and half took the IAT test after the prime. All participants made donations to charities supporting girls or boys (see below) immediately after the experimental prime. After these tasks all participants were asked to fill out an online survey (see below).

This study was approved by the Office of research and Regulatory Affairs, Rutgers University. Informed consent was received from all participants and all experiments were performed in accordance with relevant guidelines and regulations provided by the Rutgers Institutional Review Board.

### Experimental Prime

Two randomly assigned groups of participants were primed to feel either poor (the words top, best and most were used in the script below) or wealthy (the words bottom, worst and least were used in the script below) by asking them to read the following script that we copied from an experiment priming people on social class^68^.

“Think of the ladder below as representing where people stand in the United States. At the top/bottom are the people who are the best/worst off— those who have the most/least money, most/least education, and the most/least respected jobs. In particular, we’d like you to think about how YOU ARE DIFFERENT FROM THESE PEOPLE in terms of your own income, educational history, influence and job status.”

Then each of these groups (primed to feel poor or primed to feel rich) were asked to write about how they felt:

“Now imagine yourself in a getting acquainted interaction with one of the people you just thought about from the ladder above. Think about how the DIFFERENCES BETWEEN YOU might impact what you would talk about, how the interaction is likely to go, and what you and the other person might say to each other. Please write 5 complete sentences about how you think this interaction would go”.

As a control, a third group was not primed with any script and was simply asked to “Please write 5 complete sentences about today’s weather where you live”.

### Predictors

The independent variables used were designed to assess the socio-economic condition of the participants in childhood and adulthood. For clarity and simplicity all scores were coded such that higher scores indicate higher status or condition and lower values indicated lower status or condition.

#### Sex

For the statistical analyses, we coded female participants as 0 and male participants as 1.

*Childhood poverty* was assessed by consolidating responses to survey questions on whether participants had received Medicaid, benefits for low income families (AFDC, TANF, “welfare”), SNAP (food stamps), free school lunches, lived in public housing or experienced homelessness, eviction or hunger when they were growing up. Each yes response provided a participant with one negative point such that lower scores indicated more poverty in childhood.

*Current poverty* was assessed by consolidating responses to survey questions on whether participants currently receive Medicaid, benefits for low income families (AFDC, TANF, welfare), SNAP (food stamps), or live in public housing. Each yes response provided a participant with one negative point such that lower scores indicated more poverty currently.

*Income* was assessed as the approximate annual income of the participant’s household in the following ranges: (1) less than $20,000, (2) $20,000–45,000, (3) $45,001–$70,000, (4) $70,001–$100,000, and (5) greater than $100,000. *Education* and *Parents*’ *education* were assessed as the highest level of education received in the following categories: (1) less than 9th grade, (2) some high school, (3) graduated from high school, (4) some college, (5) graduated from a 2-year college, (6) graduated from a 4-year college, (7) master’s degree, (8) professional degree (e.g., law) and (9) doctoral degree.

*Perceived relative status* was assessed by asking participants to “Think of the ladder below as representing where people stand in the United States. The top rung represents people who are best off and the bottom rung represents those who are worst off.” Participants were then asked to “Please click on ONLY ONE area between the rungs that you think best represents where you stand in relation to other people.” The rungs were ranked 1–10 with 1 at the bottom and ten at the top. We coded participants’ clicks between the rungs as 0.5 at the low end and 9.5 at the high end.

*Health* was assessed by asking participants “How would you rate your own health?” and then providing them with five options: poor, fair, good, very good, and excellent.

#### Marital status

Single, married, divorced, separated, or widowed.

#### Children

Dummy coded as 0 = did not have any children, 1 = had at least one child.

### Dependent variables

Outcome variables were designed to assess preferred sex ratios and sex biased investment preferences of the participants. Outcome variables expressing a preference for boys have higher values and those expressing a preference for girls have lower values.

*Donations* to charities supporting girls or boys was expected to assess investment preferences and was obtained by asking participants the following: “In addition to the $1.50 that you will earn from participating in this experiment, you also have the opportunity to donate an additional $1 to a charity that benefits needy infants. How would you like to allocate this donation? Try to imagine that these children are your own.” The choices (their order was randomly generated) were as follows: (a) 0 cents to a boy and $1.00 to a girl, (b) 20 cents to a boy and 0.80 cents to a girl (c) 40 cents to a boy and 60 cents to a girl (d) 60 cents to a boy and 40 cents to a girl (e) 80 cents to a boy and 20 cents to a girl or (f) $1.00 to a boy and 0 cents to a girl. The outcome variable indicates the percentage donated to boys (i.e., higher numbers equal preference for boy charities). When data collection was complete, we did donate the allocated funds to Save the Children. However, contrary to what we told our participants, those funds were not directed preferentially at either boys or girls. On a debriefing screen at the end of the study, we made participants aware of this difference between what they were told regarding the donations and the reality of the situation.

*Adoption preference* (0 = girl, 1 = boy) was expected to assess sex ratio preferences and was assessed with a forced choice question “Imagine that you and your partner want to adopt a child. You visit an orphanage and pay the adoption fee. The orphanage only allows couples to adopt one child. You are given the choice of fraternal (non-identical) twins, one boy and one girl. Both are 12 months old. Whom do you choose to adopt?”

*Implicit Association Tests* were created to analyze the timed associations of positive and negative words with boy and girls words. These tests are designed to assess less deliberative and more automatic processing than self-reports^69^ and are seen to be less influenced by the desire for enhancement or social desirability^70^. It is unclear whether these tests assess sex ratio preferences, investment preferences or both. Boy words were “Masculine”, “Son”, “Male”, “Brother”, “He” and “Father” and girl words were “Feminine”, “Daughter”, “Female”, “Sister”, “She” and “Mother”. Positive words were “Healthy”, “Alive”, “Good”, “Attractive”, “Superior” and “Fertile” and negative words were “Sick”, “Dead”, “Bad”, “Ugly”, “Inferior” and “Childless”. Positive values indicated faster association times between boy words and positive words and or girl words and negative words suggesting an implicit preference for boys. Meanwhile negative indicated faster association times between boy words and negative words and or girl words and positive words suggesting an implicit preference for girls. The IAT test we developed and used can be found at: http://lynch-server.utu.fi/.

*Preferred sex ratio* preferences were expected to determine the explicit sex ratio preferences of participants and were assessed by asking:

“If you could choose the number and sex of all of the children you will have in your lifetime: How many boys would you want?— and How many girls would you want?—

The survey can be found by clicking the following link: https://missouri.qualtrics.com/jfe/preview/SV_4YIeMj7WkbyP7JX [Qualtric Survey].

## Electronic supplementary material

Supplementary Materials
