# Supplementary material for: Sexual conflict and the Trivers-Willard hypothesis: Females prefer daughters and males prefer sons
Source: Sci Rep. 2018 Oct 18;8:15463. doi: 10.1038/s41598-018-33650-1 (PMC6193998; doi:10.1038/s41598-018-33650-1)
Supplement: Supplementary file 1 — Supplementary Materials [file 41598_2018_33650_MOESM1_ESM.pdf]

Supplementary Information Tables for "Sexual conflict and the Trivers-Willard hypothesis: Females prefer daughters and males prefer sons"

Authors: Robert Lynch, Helen Wasielewski, and Lee Cronk

Table S1: Adoption model all subjects

	(Intercept)	childhood_	current_po	education_	health	income_lev	ladder	parents_ed
1320	0.380769	0.005987	0.049508	-0.02178	NA	NA	0.016085	NA
1288	0.494329	0.004808	0.044194	-0.02567	NA	NA	NA	NA
1286	0.468158	0.007415	NA	-0.0233	NA	NA	NA	NA
1316	0.244837	0.003636	0.045244	NA	NA	NA	0.019638	NA
264	0.510176	0.024401	0.047175	-0.02513	NA	NA	NA	NA
1318	0.368387	0.00869	NA	-0.01973	NA	NA	0.013751	NA
296	0.405215	0.026462	0.052269	-0.02148	NA	NA	0.014977	NA
1314	0.245219	0.00633	NA	NA	NA	NA	0.01718	NA
262	0.483136	0.028441	NA	-0.02255	NA	NA	NA	NA
292	0.270843	0.02389	0.04803	NA	NA	NA	0.018496	NA
1282	0.346605	0.004103	NA	NA	NA	NA	NA	NA
1384	0.354469	0.005107	0.048638	-0.02458	NA	NA	0.016118	0.008249
1352	0.468453	0.003932	0.04332	-0.02846	NA	NA	NA	0.008188
3368	0.384568	0.004927	0.067416	-0.02215	NA	NA	0.016713	NA
1350	0.440873	0.006417	NA	-0.02635	NA	NA	NA	0.008811
1284	0.358321	0.001604	0.037522	NA	NA	NA	NA	NA
3336	0.501225	0.003882	0.059022	-0.02611	NA	NA	NA	NA
1416	0.490498	0.004001	0.044463	-0.02504	NA	NA	NA	NA
1448	0.378091	0.005213	0.049718	-0.02122	NA	NA	0.015951	NA

prime_neg	sex	yob	childhood_	current_po	df	logLik	AICc	delta
NA	0.173773	NA	0.03943	NA	8	-534.12	1084.43	0
NA	0.174196	NA	0.037453	NA	7	-535.149	1084.445	0.015027
NA	0.182421	NA	0.039677	NA	6	-536.272	1084.653	0.223393
NA	0.172173	NA	0.038938	NA	7	-535.392	1084.932	0.501653
NA	0.142647	NA	NA	NA	6	-536.424	1084.959	0.528794
NA	0.182905	NA	0.041595	NA	7	-535.51	1085.168	0.737949
NA	0.140703	NA	NA	NA	7	-535.533	1085.212	0.782057
NA	0.180726	NA	0.040975	NA	6	-536.559	1085.229	0.798603
NA	0.149455	NA	NA	NA	5	-537.704	1085.487	1.056872
NA	0.139532	NA	NA	NA	6	-536.765	1085.64	1.210485
NA	0.179576	NA	0.038318	NA	5	-537.799	1085.677	1.247188
NA	0.172592	NA	0.038598	NA	9	-533.829	1085.895	1.464544
NA	0.173024	NA	0.036623	NA	8	-534.862	1085.914	1.484202
NA	0.165307	NA	0.043714	-0.04267	9	-533.865	1085.966	1.535874
NA	0.180985	NA	0.038736	NA	7	-535.94	1086.028	1.597663
NA	0.172347	NA	0.036313	NA	6	-536.982	1086.075	1.644548
NA	0.167117	NA	0.040977	-0.03574	8	-534.969	1086.127	1.696938
-0.01283	0.174243	NA	0.03843	NA	8	-534.981	1086.151	1.721454
-0.01215	0.173821	NA	0.040338	NA	9	-533.97	1086.176	1.746125

weight
0.012525
0.012431
0.011201
0.009746
0.009615
0.00866
0.008471
0.008401
0.007384
0.006838
0.006713
0.006022
0.005963
0.005811
0.005634
0.005504
0.005361
0.005296
0.005231

Table S2: Adoption model females

	(Intercept)	childhood_	current_po	education_	health	income_lev	ladder	parents_ec
7	0.563358	NA	0.065225	-0.03855	NA	NA	NA	NA
263	6.433518	NA	0.067752	-0.04134	NA	NA	NA	NA
135	0.556125	NA	0.063238	-0.03727	NA	NA	NA	NA
5	0.502507	NA	NA	-0.03135	NA	NA	NA	NA
391	6.463567	NA	0.065765	-0.04007	NA	NA	NA	NA
1	0.342939	NA	NA	NA	NA	NA	NA	NA
133	0.496739	NA	NA	-0.03022	NA	NA	NA	NA
261	5.930903	NA	NA	-0.03368	NA	NA	NA	NA
3	0.361498	NA	0.049921	NA	NA	NA	NA	NA
15	0.531003	NA	0.069218	-0.03798	0.012997	NA	NA	NA
39	0.520006	NA	0.068929	-0.03759	NA	NA	0.006624	NA
8	0.57227	0.005459	0.063803	-0.03944	NA	NA	NA	NA
129	0.342939	NA	NA	NA	NA	NA	NA	NA
23	0.56708	NA	0.066477	-0.03823	NA	-0.00182	NA	NA
71	0.564055	NA	0.065286	-0.03842	NA	NA	NA	-0.00027
389	5.978995	NA	NA	-0.03256	NA	NA	NA	NA
131	0.360894	NA	0.048296	NA	NA	NA	NA	NA
295	6.41514	NA	0.071651	-0.04035	NA	NA	0.006951	NA
271	6.307597	NA	0.070981	-0.04083	0.010648	NA	NA	NA

prime_neg	yob	df	logLik	AICc	delta	weight
NA	NA	4	-231.017	470.1505	0	0.027189
NA	-0.00296	5	-230.203	470.5826	0.43209	0.021906
-0.0349	NA	5	-230.398	470.9718	0.821324	0.018032
NA	NA	3	-232.524	471.1186	0.968085	0.016756
-0.03519	-0.00298	6	-229.571	471.3894	1.238896	0.014634
NA	NA	2	-233.825	471.6856	1.53512	0.01262
-0.03742	NA	4	-231.817	471.7514	1.600925	0.012211
NA	-0.00274	4	-231.832	471.7819	1.631369	0.012027
NA	NA	3	-232.91	471.8905	1.740028	0.011391
NA	NA	5	-230.925	472.0269	1.876426	0.01064
NA	NA	5	-230.944	472.0644	1.913859	0.010443
NA	NA	5	-230.965	472.1054	1.954934	0.01023
-0.03982	NA	3	-233.028	472.1265	1.975977	0.010123
NA	NA	5	-231.014	472.2039	2.053383	0.009739
NA	NA	5	-231.017	472.2092	2.058684	0.009713
-0.03778	-0.00276	5	-231.109	472.3934	2.242894	0.008858
-0.03833	NA	4	-232.169	472.4552	2.304686	0.008589
NA	-0.00297	6	-230.123	472.4931	2.342626	0.008428
NA	-0.00291	6	-230.142	472.531	2.380549	0.008269

Table S3: Adoption model males

	(Intercept)	childhood_	current_po	education_	health	income_lev	ladder	parents_ed
34	0.375629	0.049757	NA	NA	NA	NA	0.025804	NA
290	-5.13911	0.051172	NA	NA	NA	NA	0.024126	NA
42	0.421487	0.046316	NA	NA	-0.0299	NA	0.029791	NA
98	0.30008	0.045902	NA	NA	NA	NA	0.026495	0.014008
2	0.526181	0.042421	NA	NA	NA	NA	NA	NA
258	-5.82452	0.044596	NA	NA	NA	NA	NA	NA
298	-4.57931	0.047926	NA	NA	-0.02705	NA	0.027891	NA
36	0.376913	0.047045	0.023197	NA	NA	NA	0.026059	NA
50	0.325816	0.048577	NA	NA	NA	0.011168	0.029133	NA
38	0.42869	0.050931	NA	-0.00816	NA	NA	0.024177	NA
162	0.373379	0.049617	NA	NA	NA	NA	0.026252	NA
106	0.348444	0.042819	NA	NA	-0.0287	NA	0.030284	0.013205
354	-4.51212	0.047887	NA	NA	NA	NA	0.024893	0.011294
6	0.61054	0.045588	NA	-0.0158	NA	NA	NA	NA
66	0.461645	0.038764	NA	NA	NA	NA	NA	0.012641
292	-5.06466	0.048643	0.021471	NA	NA	NA	0.024385	NA
418	-5.34825	0.051039	NA	NA	NA	NA	0.02466	NA
306	-5.09934	0.050079	NA	NA	NA	0.010134	0.027173	NA
46	0.474507	0.047489	NA	-0.00816	-0.02989	NA	0.028165	NA
294	-5.02768	0.052232	NA	-0.00748	NA	NA	0.022655	NA

prime_neg	yob	df	logLik	AICc	delta	weight
NA	NA	4	-302.017	612.1299	0	0.036199
NA	0.002789	5	-301.363	612.8693	0.739425	0.025011
NA	NA	5	-301.37	612.8849	0.754983	0.024817
NA	NA	5	-301.56	613.2637	1.133849	0.020535
NA	NA	3	-303.635	613.328	1.198102	0.019885
NA	0.003206	4	-302.768	613.6312	1.501312	0.017088
NA	0.002527	6	-300.838	613.8774	1.747491	0.015109
NA	NA	5	-301.893	613.9293	1.79943	0.014722
NA	NA	5	-301.899	613.942	1.812169	0.014628
NA	NA	5	-301.921	613.986	1.856089	0.01431
0.010441	NA	5	-301.96	614.0633	1.933462	0.013767
NA	NA	6	-300.964	614.1301	2.000222	0.013315
NA	0.002441	6	-301.075	614.3518	2.22189	0.011918
NA	NA	4	-303.254	614.6033	2.473408	0.01051
NA	NA	4	-303.265	614.6258	2.495881	0.010393
NA	0.002752	6	-301.256	614.7138	2.583926	0.009945
0.013904	0.002893	6	-301.262	614.7251	2.595176	0.009889
NA	0.002746	6	-301.265	614.7327	2.602841	0.009851
NA	NA	6	-301.274	614.7505	2.620593	0.009764
NA	0.002757	6	-301.282	614.7657	2.635807	0.00969

Table S4: Donation models all subjects

	(Intercept)	childhood_	current_po	education_	health	income_lev	ladder	parents_ed
12258	0.919652	-0.00129	NA	NA	NA	NA	-0.00122	-0.00833
16354	0.876026	-0.00078	NA	NA	NA	NA	-0.00136	-0.00849
11234	0.672466	0.006178	NA	NA	NA	NA	-0.00025	-0.008
12260	0.872188	-0.0008	-0.00939	NA	NA	NA	-0.00233	-0.00816
11233	0.939186	NA	NA	NA	NA	NA	-0.00089	-0.00691
12274	0.898549	-0.0012	NA	NA	NA	-0.00344	-0.00251	-0.00804
12262	1.023126	-0.00105	NA	-0.00287	NA	NA	-0.00155	-0.0077
15330	0.633009	0.006504	NA	NA	NA	NA	-0.00043	-0.00818
12266	0.933267	-0.00134	NA	NA	-0.001	NA	-0.00112	-0.00833
16356	0.832549	-0.00033	-0.00891	NA	NA	NA	-0.00241	-0.00832
15329	0.916115	NA	NA	NA	NA	NA	-0.00109	-0.00702
11236	0.623705	0.006846	-0.00801	NA	NA	NA	-0.00116	-0.00784
16358	0.976969	-0.00055	NA	-0.00279	NA	NA	-0.00168	-0.00788
16370	0.856363	-0.00071	NA	NA	NA	-0.0033	-0.00259	-0.00821
11238	0.77766	0.00641	NA	-0.0029	NA	NA	-0.00059	-0.00737
16362	0.888108	-0.00083	NA	NA	-0.00088	NA	-0.00128	-0.00849
11250	0.65094	0.006341	NA	NA	NA	-0.00301	-0.00136	-0.00774
12264	0.964637	-0.00061	-0.00894	-0.0025	NA	NA	-0.00256	-0.00762
12276	0.86461	-0.0008	-0.00835	NA	NA	-0.0021	-0.00299	-0.008
11242	0.696275	0.006031	NA	NA	-0.00162	NA	-0.0001	-0.008

prime_neg	sex	yob	childhood_	ladder:sex	prime_neg	sex:yob	df	logLik
-0.01702	-6.26682	-0.00024	0.01433	0.020129	NA	0.003142	11	309.9389
-0.00825	-6.116	-0.00022	0.013943	0.019962	-0.01649	0.003066	12	310.5561
-0.01621	-5.86729	-0.00012	NA	0.017862	NA	0.002941	10	308.3447
-0.0168	-6.2457	-0.00022	0.014894	0.021144	NA	0.003129	12	310.3799
-0.01658	-5.9412	-0.00025	NA	0.017398	NA	0.00298	9	307.2049
-0.01711	-6.24144	-0.00022	0.014535	0.020396	NA	0.003128	12	310.122
-0.01664	-6.29831	-0.00029	0.014309	0.019954	NA	0.003158	12	310.1202
-0.00687	-5.71775	-9.56E-05	NA	0.017749	-0.01761	0.002866	11	309.0473
-0.01698	-6.2602	-0.00025	0.014266	0.020175	NA	0.003138	12	309.9503
-0.00835	-6.10137	-0.00019	0.014492	0.020931	-0.0159	0.003056	13	310.9531
-0.00809	-5.80869	-0.00024	NA	0.017274	-0.01602	0.002914	10	307.7874
-0.016	-5.83588	-9.08E-05	NA	0.018651	NA	0.002923	11	308.6657
-0.00795	-6.14782	-0.00026	0.013925	0.019794	-0.01636	0.003083	13	310.7278
-0.00844	-6.09357	-0.0002	0.014144	0.02022	-0.01629	0.003054	13	310.7241
-0.01583	-5.89979	-0.00016	NA	0.017688	NA	0.002958	11	308.5301
-0.00823	-6.11046	-0.00022	0.013887	0.020003	-0.01646	0.003063	13	310.565
-0.01628	-5.84013	-9.89E-05	NA	0.018066	NA	0.002927	11	308.4842
-0.01648	-6.27416	-0.00026	0.014848	0.020942	NA	0.003144	13	310.5166
-0.01687	-6.23259	-0.00021	0.014956	0.021193	NA	0.003122	13	310.4425
-0.01616	-5.85946	-0.00013	NA	0.017952	NA	0.002937	11	308.3746

AICc	delta	weight
-597.529	0	0.038577
-596.7	0.829331	0.025482
-596.4	1.129795	0.021928
-596.348	1.181882	0.021364
-596.173	1.356413	0.019579
-595.832	1.697504	0.016509
-595.828	1.701091	0.016479
-595.746	1.783148	0.015817
-595.488	2.041028	0.013904
-595.425	2.104753	0.013468
-595.285	2.244542	0.012558
-594.983	2.546364	0.010799
-594.974	2.555333	0.010751
-594.967	2.562645	0.010712
-594.712	2.817453	0.00943
-594.649	2.880899	0.009136
-594.62	2.909227	0.009007
-594.552	2.977642	0.008704
-594.403	3.125958	0.008082
-594.401	3.12851	0.008072

Table S5: Donation models females

	(Intercept)	childhood_	current_po	education_	health	income_lev	ladder	parents_ed
69	0.497593	NA	NA	-0.01124	NA	NA	NA	-0.00923
65	0.454813	NA	NA	NA	NA	NA	NA	-0.01225
197	0.496719	NA	NA	-0.01096	NA	NA	NA	-0.00933
5	0.476131	NA	NA	-0.01564	NA	NA	NA	NA
101	0.521674	NA	NA	-0.01187	NA	NA	-0.00336	-0.00936
85	0.492929	NA	NA	-0.01204	NA	0.003736	NA	-0.00948
325	1.160526	NA	NA	-0.01175	NA	NA	NA	-0.00875
70	0.501382	0.001831	NA	-0.01152	NA	NA	NA	-0.00938
77	0.489842	NA	NA	-0.01099	0.002842	NA	NA	-0.00929
71	0.500876	NA	0.003213	-0.01153	NA	NA	NA	-0.00935
193	0.454999	NA	NA	NA	NA	NA	NA	-0.01229
73	0.443396	NA	NA	NA	0.004768	NA	NA	-0.01224
97	0.467209	NA	NA	NA	NA	NA	-0.00192	-0.01243
261	1.69106	NA	NA	-0.01616	NA	NA	NA	NA
321	0.662239	NA	NA	NA	NA	NA	NA	-0.01215
66	0.455816	0.000675	NA	NA	NA	NA	NA	-0.01234
81	0.453003	NA	NA	NA	NA	0.000874	NA	-0.01236
67	0.454677	NA	-0.0002	NA	NA	NA	NA	-0.01224
133	0.475092	NA	NA	-0.01543	NA	NA	NA	NA
229	0.519538	NA	NA	-0.01157	NA	NA	-0.00318	-0.00946

prime_neg	yob	df	logLik	AICc	delta	weight
NA	NA	4	175.8758	-343.635	0	0.045367
NA	NA	3	174.3444	-342.619	1.015768	0.0273
-0.0073	NA	5	176.1584	-342.141	1.493864	0.021496
NA	NA	3	174.0488	-342.028	1.607064	0.020313
NA	NA	5	176.084	-341.992	1.642603	0.019955
NA	NA	5	176.0226	-341.869	1.765453	0.018766
NA	-0.00033	5	175.9797	-341.783	1.851266	0.017978
NA	NA	5	175.9373	-341.699	1.936076	0.017231
NA	NA	5	175.9241	-341.672	1.962379	0.017006
NA	NA	5	175.9138	-341.652	1.982971	0.016832
-0.00824	NA	4	174.7024	-341.288	2.346798	0.014033
NA	NA	4	174.4813	-340.846	2.788971	0.011249
NA	NA	4	174.4135	-340.71	2.92474	0.010511
NA	-0.00061	4	174.4094	-340.702	2.932784	0.010469
NA	-0.00011	4	174.3549	-340.593	3.041858	0.009913
NA	NA	4	174.3529	-340.589	3.045927	0.009893
NA	NA	4	174.3528	-340.589	3.045987	0.009893
NA	NA	4	174.3446	-340.572	3.062452	0.009811
-0.00674	NA	4	174.2871	-340.457	3.177384	0.009264
-0.00701	NA	6	176.3446	-340.442	3.19249	0.009194

Table S6: Donations models males

	(Intercept)	childhood_	current_po	education_	health	income_lev	ladder	parents_ed
418	-4.78678	0.010682	NA	NA	NA	NA	0.019225	NA
420	-4.85755	0.012634	-0.01664	NA	NA	NA	0.019058	NA
482	-5.05473	0.01215	NA	NA	NA	NA	0.018856	-0.00502
417	-4.56132	NA	NA	NA	NA	NA	0.017107	NA
434	-4.81088	0.011368	NA	NA	NA	-0.00635	0.017313	NA
426	-4.68879	0.010087	NA	NA	-0.00494	NA	0.019922	NA
422	-4.8254	0.010267	NA	0.002957	NA	NA	0.019792	NA
484	-5.13062	0.014151	-0.01687	NA	NA	NA	0.018681	-0.0051
425	-4.42555	NA	NA	NA	-0.00784	NA	0.018404	NA
290	-5.14052	0.010457	NA	NA	NA	NA	0.020128	NA
428	-4.73952	0.012046	-0.01805	NA	-0.00625	NA	0.019927	NA
421	-4.63654	NA	NA	0.004694	NA	NA	0.018139	NA
486	-5.15723	0.011744	NA	0.004562	NA	NA	0.019673	-0.00583
436	-4.86658	0.012899	-0.0145	NA	NA	-0.00477	0.017642	NA
498	-5.04921	0.012588	NA	NA	NA	-0.00538	0.017273	-0.00454
424	-4.90033	0.012218	-0.01691	0.003187	NA	NA	0.019667	NA
490	-4.95608	0.011548	NA	NA	-0.00519	NA	0.019584	-0.0051
433	-4.56755	NA	NA	NA	NA	-0.0041	0.015784	NA
481	-4.67433	NA	NA	NA	NA	NA	0.016793	-0.00239
442	-4.6901	0.010707	NA	NA	-0.00625	-0.00721	0.017938	NA

prime_neg	yob	df	logLik	AICc	delta	weight
-0.02352	0.002595	6	141.202	-270.202	0	0.069342
-0.02265	0.002631	7	141.7192	-269.169	1.033628	0.041357
-0.02424	0.002745	7	141.6612	-269.052	1.149721	0.039024
-0.02317	0.002484	5	139.5694	-268.995	1.207243	0.037918
-0.02357	0.002622	7	141.5119	-268.754	1.448207	0.033614
-0.02324	0.00255	7	141.3433	-268.417	1.78546	0.028398
-0.02388	0.002605	7	141.3039	-268.338	1.864243	0.027301
-0.02337	0.002783	8	142.1937	-268.04	2.162518	0.023519
-0.02276	0.002421	6	139.9364	-267.671	2.531291	0.019558
NA	0.002772	5	138.8703	-267.597	2.605387	0.018847
-0.02222	0.002576	8	141.9424	-267.537	2.665242	0.018291
-0.02376	0.002507	6	139.8303	-267.459	2.743494	0.01759
-0.02491	0.002784	8	141.8923	-267.437	2.765316	0.017399
-0.0228	0.002646	8	141.8861	-267.424	2.777856	0.01729
-0.02421	0.002753	8	141.8797	-267.412	2.790648	0.01718
-0.02302	0.002642	8	141.8377	-267.328	2.874643	0.016473
-0.02396	0.002699	8	141.8177	-267.288	2.914564	0.016148
-0.02319	0.002497	6	139.7006	-267.199	3.002916	0.01545
-0.02349	0.002548	6	139.6799	-267.158	3.044283	0.015133
-0.02323	0.002568	8	141.7329	-267.118	3.084099	0.014835

Table S7: Implicit Association Test models all subjects

	(Intercept)	childhood_	current_po	education_	income_lev	ladder	parents_ec	prime_neg
225	-0.35875	NA	NA	NA	NA	NA	-0.01678	-0.03602
227	-0.34096	NA	0.03171	NA	NA	NA	-0.01806	-0.03651
161	-0.36125	NA	NA	NA	NA	NA	-0.01614	NA
193	-0.43902	NA	NA	NA	NA	NA	NA	-0.03459
129	-0.43846	NA	NA	NA	NA	NA	NA	NA
163	-0.34403	NA	0.030744	NA	NA	NA	-0.01737	NA
241	-0.32407	NA	NA	NA	NA	-0.00534	-0.01734	-0.03641
229	-0.33704	NA	NA	-0.00546	NA	NA	-0.01552	-0.03523
226	-0.35383	0.002891	NA	NA	NA	NA	-0.01727	-0.03593
481	0.033688	NA	NA	NA	NA	NA	-0.01658	-0.03618
233	-0.35839	NA	NA	NA	-0.00018	NA	-0.01676	-0.03601
195	-0.4295	NA	0.025887	NA	NA	NA	NA	-0.0349
197	-0.38116	NA	NA	-0.01139	NA	NA	NA	-0.03318
231	-0.30785	NA	0.034305	-0.00797	NA	NA	-0.01632	-0.03541
133	-0.37341	NA	NA	-0.01281	NA	NA	NA	NA
165	-0.33187	NA	NA	-0.00737	NA	NA	-0.01445	NA
235	-0.32418	NA	0.036493	NA	-0.00694	NA	-0.01728	-0.03639
177	-0.33075	NA	NA	NA	NA	-0.0047	-0.01663	NA
131	-0.42919	NA	0.025175	NA	NA	NA	NA	NA
243	-0.32311	NA	0.030042	NA	NA	-0.00289	-0.0183	-0.0367

sex	yob	df	logLik	AICc	delta	weight
0.549348	NA	5	-410.847	831.7828	0	0.040108
0.544465	NA	6	-410.203	832.5312	0.748411	0.027588
0.550957	NA	4	-412.254	832.5679	0.785159	0.027085
0.546569	NA	4	-412.301	832.6602	0.877382	0.025865
0.548219	NA	3	-413.595	833.2261	1.443284	0.019491
0.546245	NA	5	-411.652	833.3922	1.60941	0.017937
0.549797	NA	6	-410.718	833.5602	1.77741	0.016492
0.549938	NA	6	-410.762	833.6479	1.865133	0.015784
0.548959	NA	6	-410.815	833.7548	1.97205	0.014962
0.549557	-0.0002	6	-410.84	833.8039	2.021118	0.0146
0.54932	NA	6	-410.847	833.8183	2.035537	0.014495
0.54241	NA	5	-411.868	833.8246	2.041793	0.01445
0.548236	NA	5	-411.896	833.88	2.097247	0.014055
0.544927	NA	7	-410.026	834.2183	2.435502	0.011868
0.550019	NA	4	-413.082	834.2238	2.440991	0.011835
0.551707	NA	5	-412.099	834.2859	2.503147	0.011473
0.542632	NA	7	-410.103	834.3727	2.589891	0.010986
0.551368	NA	5	-412.155	834.3982	2.615375	0.010847
0.544189	NA	4	-413.188	834.4344	2.651605	0.010652
0.544966	NA	7	-410.167	834.5006	2.717775	0.010306

Table S8: Implicit Association Test models females

	(Intercept)	childhood_	current_po	education_	income_lev	ladder	parents_ec	prime_neg
1	-0.43846	NA	NA	NA	NA	NA	NA	NA
11	-0.30837	NA	0.066272	NA	-0.03909	NA	NA	NA
65	-0.43904	NA	NA	NA	NA	NA	NA	-0.03586
2	-0.45372	-0.01709	NA	NA	NA	NA	NA	NA
75	-0.30634	NA	0.065378	NA	-0.04019	NA	NA	-0.03674
33	-0.36662	NA	NA	NA	NA	NA	-0.01501	NA
9	-0.37986	NA	NA	NA	-0.02167	NA	NA	NA
12	-0.3272	-0.01897	0.072484	NA	-0.03755	NA	NA	NA
3	-0.42536	NA	0.035603	NA	NA	NA	NA	NA
66	-0.45632	-0.01929	NA	NA	NA	NA	NA	-0.03979
76	-0.32706	-0.02109	0.072188	NA	-0.03858	NA	NA	-0.04067
43	-0.24401	NA	0.070259	NA	-0.03582	NA	-0.015	NA
5	-0.36107	NA	NA	-0.01524	NA	NA	NA	NA
73	-0.37679	NA	NA	NA	-0.02304	NA	NA	-0.03766
4	-0.44065	-0.02043	0.043602	NA	NA	NA	NA	NA
97	-0.36472	NA	NA	NA	NA	NA	-0.01554	-0.03688
35	-0.33651	NA	0.043457	NA	NA	NA	-0.01796	NA
15	-0.24346	NA	0.069813	-0.0145	-0.03538	NA	NA	NA
67	-0.42653	NA	0.033948	NA	NA	NA	NA	-0.0347
107	-0.24051	NA	0.069438	NA	-0.03686	NA	-0.01533	-0.03735

yob	df	logLik	AICc	delta	weight
NA	2	-176.943	357.9247	0	0.024657
NA	4	-175.04	358.2134	0.288606	0.021344
NA	3	-176.251	358.5815	0.656773	0.017755
NA	3	-176.364	358.8063	0.881585	0.015867
NA	5	-174.308	358.8149	0.890197	0.015799
NA	3	-176.38	358.8394	0.914619	0.015607
NA	3	-176.399	358.8781	0.95337	0.015308
NA	5	-174.342	358.8836	0.958868	0.015266
NA	3	-176.452	358.9825	1.057797	0.014529
NA	4	-175.518	359.1693	1.244593	0.013234
NA	6	-173.449	359.1771	1.252365	0.013182
NA	5	-174.499	359.1974	1.272638	0.013049
NA	3	-176.607	359.2922	1.36748	0.012445
NA	4	-175.636	359.4052	1.480464	0.011761
NA	4	-175.646	359.4245	1.499729	0.011649
NA	4	-175.647	359.4256	1.500819	0.011642
NA	4	-175.667	359.4668	1.542038	0.011405
NA	5	-174.761	359.7221	1.797395	0.010038
NA	4	-175.804	359.7396	1.814813	0.009951
NA	6	-173.739	359.7589	1.834109	0.009855

Table S9: Implicit Association Test models males

	(Intercept)	childhood_	current_po	education_	income_lev	ladder	parents_ec	prime_neg
1	0.109759	NA	NA	NA	NA	NA	NA	NA
33	0.194385	NA	NA	NA	NA	NA	-0.01708	NA
65	0.107617	NA	NA	NA	NA	NA	NA	-0.03347
34	0.230822	0.020573	NA	NA	NA	NA	-0.02141	NA
97	0.195795	NA	NA	NA	NA	NA	-0.01782	-0.03529
2	0.120292	0.014468	NA	NA	NA	NA	NA	NA
98	0.234651	0.021881	NA	NA	NA	NA	-0.02248	-0.03784
5	0.16682	NA	NA	-0.01093	NA	NA	NA	NA
17	0.142851	NA	NA	NA	NA	-0.00545	NA	NA
9	0.090314	NA	NA	NA	0.007572	NA	NA	NA
66	0.11873	0.015394	NA	NA	NA	NA	NA	-0.03494
3	0.112162	NA	0.011552	NA	NA	NA	NA	NA
129	0.221648	NA	NA	NA	NA	NA	NA	NA
41	0.170041	NA	NA	NA	0.013166	NA	-0.01899	NA
49	0.245403	NA	NA	NA	NA	-0.00765	-0.018	NA
37	0.220568	NA	NA	-0.00613	NA	NA	-0.01591	NA
35	0.198596	NA	0.014466	NA	NA	NA	-0.01732	NA
161	-0.64664	NA	NA	NA	NA	NA	-0.01749	NA
6	0.202197	0.017654	NA	-0.01525	NA	NA	NA	NA
69	0.15514	NA	NA	-0.00909	NA	NA	NA	-0.03186

yob	df	logLik	AICc	delta	weight
NA	2	-236.173	476.3776	0	0.03755
NA	3	-235.393	476.8512	0.473604	0.029633
NA	3	-235.557	477.1783	0.800718	0.025162
NA	4	-234.605	477.3185	0.940953	0.023458
NA	4	-234.707	477.5225	1.144951	0.021183
NA	3	-235.76	477.5851	1.207507	0.020531
NA	5	-233.816	477.7944	1.416798	0.018491
NA	3	-235.971	478.0077	1.630062	0.016621
NA	3	-236.094	478.2532	1.875601	0.014701
NA	3	-236.097	478.2594	1.88178	0.014655
NA	4	-235.089	478.287	1.909397	0.014454
NA	3	-236.137	478.339	1.961419	0.014083
-5.65E-05	3	-236.172	478.4094	2.031842	0.013596
NA	4	-235.174	478.4566	2.079052	0.013279
NA	4	-235.24	478.589	2.211398	0.012428
NA	4	-235.334	478.7751	2.397542	0.011324
NA	4	-235.338	478.7831	2.405518	0.011279
0.000426	4	-235.377	478.8619	2.484295	0.010843
NA	4	-235.388	478.8838	2.506181	0.010725
NA	4	-235.419	478.9458	2.568251	0.010397

Table S10: Explicitly preferred sex ratios models all subjects

	(Intercept)	childhood_	current_po	education_	health	income_lev	parents_ec	prime_neg
193	0.481197	NA	NA	NA	NA	NA	NA	-0.01792
129	0.481197	NA	NA	NA	NA	NA	NA	NA
133	0.526682	NA	NA	-0.00898	NA	NA	NA	NA
197	0.522127	NA	NA	-0.00808	NA	NA	NA	-0.01665
209	0.496447	NA	NA	NA	NA	-0.00551	NA	-0.01771
201	0.465689	NA	NA	NA	0.006544	NA	NA	-0.01807
195	0.47756	NA	-0.00974	NA	NA	NA	NA	-0.01755
145	0.497291	NA	NA	NA	NA	-0.00582	NA	NA
131	0.477139	NA	-0.01087	NA	NA	NA	NA	NA
137	0.466449	NA	NA	NA	0.006223	NA	NA	NA
194	0.481799	0.000645	NA	NA	NA	NA	NA	-0.01791
225	0.478663	NA	NA	NA	NA	NA	0.000537	-0.0179
161	0.478238	NA	NA	NA	NA	NA	0.000627	NA
130	0.481826	0.000674	NA	NA	NA	NA	NA	NA
165	0.518097	NA	NA	-0.01011	NA	NA	0.003029	NA
135	0.520944	NA	-0.00796	-0.00844	NA	NA	NA	NA
141	0.514035	NA	NA	-0.00858	0.004473	NA	NA	NA
134	0.530678	0.002159	NA	-0.00937	NA	NA	NA	NA
149	0.530169	NA	NA	-0.00821	NA	-0.00267	NA	NA
205	0.508034	NA	NA	-0.00762	0.004967	NA	NA	-0.01683

sex	df	logLik	AICc	delta	weight
0.044323	4	9.815359	-11.5649	0	0.046847
0.045794	3	8.711323	-11.3832	0.181693	0.042779
0.046999	4	9.517094	-10.9684	0.59653	0.034765
0.045512	5	10.46446	-10.8301	0.734849	0.032442
0.043728	5	10.04414	-9.98944	1.575487	0.021309
0.044324	5	10.00518	-9.91151	1.653419	0.020495
0.046006	5	10.004	-9.90916	1.655769	0.020471
0.045148	4	8.965401	-9.86501	1.699916	0.020024
0.047637	4	8.945878	-9.82597	1.738963	0.019637
0.045806	4	8.882428	-9.69907	1.865863	0.01843
0.044179	5	9.820125	-9.5414	2.023526	0.017033
0.044234	5	9.819883	-9.54092	2.02401	0.017028
0.045688	4	8.717469	-9.36915	2.19578	0.015627
0.045642	4	8.716511	-9.36723	2.197695	0.015612
0.04664	5	9.648326	-9.19781	2.367123	0.014344
0.048276	5	9.640122	-9.1814	2.383532	0.014227
0.046954	5	9.604075	-9.1093	2.455626	0.013723
0.046566	5	9.56892	-9.03899	2.525937	0.013249
0.046599	5	9.564793	-9.03074	2.534189	0.013194
0.045445	6	10.57192	-9.00523	2.559698	0.013027

Table S11: Explicitly preferred sex ratios models females

	(Intercept)	childhood_	current_po	education_	health	income_lev	parents_ec	prime_neg
1	0.481197	NA	NA	NA	NA	NA	NA	NA
33	0.532988	NA	NA	NA	NA	NA	-0.01097	NA
17	0.525262	NA	NA	NA	NA	-0.01592	NA	NA
65	0.481197	NA	NA	NA	NA	NA	NA	-0.02301
81	0.526067	NA	NA	NA	NA	-0.01621	NA	-0.02345
97	0.531211	NA	NA	NA	NA	NA	-0.01059	-0.02216
49	0.5604	NA	NA	NA	NA	-0.01308	-0.00911	NA
3	0.475108	NA	-0.01631	NA	NA	NA	NA	NA
9	0.454057	NA	NA	NA	0.011453	NA	NA	NA
113	0.559463	NA	NA	NA	NA	-0.0135	-0.00866	-0.02268
67	0.475108	NA	-0.01631	NA	NA	NA	NA	-0.02301
73	0.451081	NA	NA	NA	0.012709	NA	NA	-0.02376
2	0.478344	-0.00306	NA	NA	NA	NA	NA	NA
41	0.507024	NA	NA	NA	0.010534	NA	-0.01076	NA
35	0.525105	NA	-0.0129	NA	NA	NA	-0.01032	NA
5	0.489985	NA	NA	-0.00174	NA	NA	NA	NA
25	0.503475	NA	NA	NA	0.008099	-0.01498	NA	NA
37	0.517146	NA	NA	0.004173	NA	NA	-0.01209	NA
66	0.477254	-0.00423	NA	NA	NA	NA	NA	-0.02381
34	0.530935	-0.00133	NA	NA	NA	NA	-0.0108	NA

df	logLik	AICc	delta	weight
2	-5.65894	15.36058	0	0.045143
3	-4.78448	15.65468	0.294098	0.03897
3	-4.81884	15.72339	0.362813	0.037653
3	-4.84844	15.7826	0.422019	0.036555
4	-3.97253	16.08844	0.727859	0.031371
4	-4.02958	16.20254	0.841961	0.029632
4	-4.2399	16.62317	1.262592	0.024011
3	-5.36505	16.81581	1.455229	0.021807
3	-5.43467	16.95506	1.594479	0.02034
5	-3.44668	17.10918	1.748605	0.018831
4	-4.55287	17.2491	1.888526	0.017559
4	-4.57155	17.28646	1.925882	0.017234
3	-5.60415	17.29401	1.933431	0.017169
4	-4.59395	17.33127	1.970695	0.016852
4	-4.60282	17.34902	1.988438	0.016703
3	-5.64695	17.37961	2.019036	0.01645
4	-4.70899	17.56136	2.200778	0.015021
4	-4.72389	17.59114	2.230564	0.014799
4	-4.74419	17.63176	2.27118	0.014501
4	-4.77428	17.69193	2.331352	0.014072

Table S12: Explicitly preferred sex ratios models males

	(Intercept)	childhood_	current_po	education_	health	income_lev	parents_ec	prime_neg
37	0.547479	NA	NA	-0.01896	NA	NA	0.015956	NA
53	0.540494	NA	NA	-0.02121	NA	0.008198	0.015332	NA
101	0.545525	NA	NA	-0.01839	NA	NA	0.015624	-0.00811
38	0.558032	0.004415	NA	-0.01959	NA	NA	0.015112	NA
5	0.601773	NA	NA	-0.01439	NA	NA	NA	NA
45	0.544207	NA	NA	-0.01887	0.001141	NA	0.015984	NA
39	0.547395	NA	-0.00019	-0.01895	NA	NA	0.015958	NA
33	0.468168	NA	NA	NA	NA	NA	0.012028	NA
1	0.526991	NA	NA	NA	NA	NA	NA	NA
21	0.58989	NA	NA	-0.01757	NA	0.010695	NA	NA
6	0.617033	0.008772	NA	-0.01613	NA	NA	NA	NA
117	0.538215	NA	NA	-0.02069	NA	0.008446	0.014961	-0.00858
54	0.549163	0.003408	NA	-0.02153	NA	0.007583	0.014727	NA
61	0.529353	NA	NA	-0.02116	0.003659	0.008962	0.015365	NA
55	0.538077	NA	-0.00453	-0.02119	NA	0.00872	0.015332	NA
97	0.468745	NA	NA	NA	NA	NA	0.01171	-0.01192
69	0.597672	NA	NA	-0.01377	NA	NA	NA	-0.01078
102	0.557177	0.004954	NA	-0.01905	NA	NA	0.014644	-0.0089
109	0.542248	NA	NA	-0.01831	0.001143	NA	0.015652	-0.00811
65	0.5259	NA	NA	NA	NA	NA	NA	-0.01329

df	logLik	AICc	delta	weight
4	18.3326	-28.5417	0	0.091699
5	18.58288	-26.98	1.561733	0.041999
5	18.45891	-26.7321	1.809687	0.037102
5	18.43113	-26.6765	1.86523	0.036086
3	16.29206	-26.5103	2.03147	0.033207
5	18.3362	-26.4866	2.055093	0.032817
5	18.33263	-26.4795	2.062239	0.0327
3	16.24803	-26.4222	2.119527	0.031777
2	15.0279	-26.019	2.522745	0.025975
4	16.71833	-25.3132	3.22854	0.018251
4	16.69909	-25.2747	3.267016	0.017904
6	18.72434	-25.1878	3.353929	0.017142
6	18.64027	-25.0197	3.522075	0.01576
6	18.61781	-24.9747	3.566997	0.01541
6	18.59957	-24.9383	3.603461	0.015131
4	16.52161	-24.9198	3.621968	0.014992
4	16.51417	-24.9049	3.636866	0.014881
6	18.58189	-24.9029	3.638826	0.014866
6	18.46252	-24.6642	3.877565	0.013193
3	15.36659	-24.6593	3.882397	0.013162

Supplementary materials captions file

Table S1: Adoption model all subjects

Top ranked models for predicting adoption preferences of all subjects ranked by AICc score from lowest to highest.

Table S2: Adoption model females

Top ranked models for predicting adoption preferences of females ranked by AICc score from lowest to highest.

Table S3: Adoption model males

Top ranked models for predicting adoption preferences of males ranked by AICc score from lowest to highest.

Table S4: Donation models all subjects

Top ranked models for predicting donations preferences of all subjects ranked by AICc score from lowest to highest.

Table S5: Donation models females

Top ranked models for predicting donations preferences of females ranked by AICc score from lowest to highest.

Table S6: Donations models males

Top ranked models for predicting donations preferences of males ranked by AICc score from lowest to highest.

Table S7: Implicit Association Test models all subjects

Top ranked models for predicting IAT preferences of all subjects ranked by AICc score from lowest to highest.

Table S8: Implicit Association Test models females

Top ranked models for predicting IAT preferences of females ranked by AICc score from lowest to highest.

Table S9: Implicit Association Test models males

Top ranked models for predicting IAT preferences of males ranked by AICc score from lowest to highest.

Table S10: Explicitly preferred sex ratios models all subjects

Top ranked models for predicting explicit sex ratio preferences of all subjects ranked by AICc score from lowest to highest.

Table S11: Explicitly preferred sex ratios models females

Top ranked models for predicting explicit sex ratio preferences of females ranked by AICc score from lowest to highest.

Table S12: Explicitly preferred sex ratios models males

Top ranked models for predicting explicit sex ratio preferences of males ranked by AICc score from lowest to highest.
